# Assessing the potential role of arbuscular mycorrhizal fungi in improving the phytochemical content and antioxidant properties in *Gomphrena globosa*

**DOI:** 10.1038/s41598-024-73479-5

**Published:** 2024-10-01

**Authors:** Rajni Dhalaria, Rachna Verma, Rohit Sharma, Klaudia Jomova, Eugenie Nepovimova, Harsh Kumar, Kamil Kuca

**Affiliations:** 1 School of Biological and Environmental Sciences, Shoolini University of Biotechnology and Management Sciences, Solan, Himachal Pradesh 173229 India; 2 Department of Chemistry, Faculty of Science, University of Hradec Kralove, Rokitanskeho 62, 50003 Hradec Kralove, Czech Republic; 3 Department of Rasa Shastra and Bhaishajya Kalpana, Faculty of Ayurveda, Institute of Medical Sciences, Banaras Hindu University, Varanasi, Uttar Pradesh 221005 India; 4 Department of Chemistry, Faculty of Natural Sciences and Informatics, Constantine the Philosopher University in Nitra, 94974 Nitra, Slovakia; 5 Centre of Advanced Technologies, Faculty of Science, University of Hradec Kralove, Rokitanskeho 62, 50003 Hradec Kralove, Czech Republic; 6 Research Institute for Biomedical Science, Antonina Dvoraka 451/1, Hradec Kralove, 500 02 Czech Republic

**Keywords:** Arbuscular mycorrhizal fungi, Nutrient uptake, Phytoconstituents, Symbiotic interaction, Antioxidant potential, *Gomphrena globosa*, Plant sciences, Plant symbiosis, Arbuscular mycorrhiza

## Abstract

Strategies to increase the secondary metabolite production, obtained from medicinal plants has been the topic of research in recent years. The symbiotic interaction between arbuscular mycorrhizal fungi and plants allows host-fungus pairings to enhance secondary metabolite synthesis. Therefore, the current study investigated the effect of inoculating two distinct AMF species discretely as well as in conjunction on the flower-derived secondary metabolites in *Gomphrena globosa*. The findings showed that the plants inoculated with combined treatment exhibited higher total phenolic (50.11 mg GAE/g DW), flavonoids (29.67 mg QE/g DW), saponins (122.55 mg DE/g DW), tannins (165.71 TAE/g DW) and terpenoid (8.24 mg LE/g DW) content in the methanolic extract. HPTLC examination showed the existence of kaempferol and benzoic acid with the highest amount (0.90% and 5.83% respectively) observed in the same treatment. FTIR analysis revealed functional group peaks with increased peak intensity in the combination treatment. Higher antioxidant activities such as DPPH (IC_50_: 401.39 µg/mL), ABTS (IC_50_: 71.18 µg/mL) and FRAP (8774.73 µM Fe (II) equivalent) were observed in the methanolic extract of combined treatment. To our knowledge, this is the first study on the impact of AMF inoculation on bioactive compounds and antioxidant activities in *G. globosa* flowers. Moreover, this study could lead to the development of novel pharmaceuticals and herbal remedies for various diseases.

**Supplementary Information:**

The online version contains supplementary material available at 10.1038/s41598-024-73479-5.

## Introduction

Plant associations with arbuscular mycorrhizal fungi (AMF) are essential for nutrient absorption and therefore, have a significant impact on productivity through increased tolerance to abiotic and biotic stress^1–3^. Several aromatic and medicinal plant species have been examined for possible health-enhancing compounds after AMF inoculation^4,5^. Increased production of secondary metabolites was observed after AMF inoculation that stimulates secondary metabolism by increasing the gene expression which drive important metabolic pathways^6^. The enhanced production of major classes of secondary metabolites including alkaloids, phenolics and terpenes via these pathways demonstrates the profound impact that AMF has on the allocation of resources to and by the host^7–10^. The aforementioned component of mycorrhizal symbiosis is very significant since mycorrhized plants have herbage with greater content of bioactive compounds, making it more appealing to the pharmaceutical sector.

AMF represent an important group of root endophytes due to their key role in the enhancement of plant nutrition, health and product quality^11,12^. Similar to the mycorrhizal fungi, endophytic fungi are known to stimulate plant growth and production of secondary metabolites by modulating the metabolic profile of their host plants^13,14^. AMF colonization with host entails a series of symbiotic interactions that are regulated by both the partners, finally leading to AMF-host compatibility. Thus, the degree of the symbiotic strength or weakness may depend on the functional characteristics of both partners^6,15^. Previous studies conducted to analyze the impact of these mycorrhizal fungi on production of plant metabolites have revealed a wide range of interactions between AMF and its hosts^16^. Numerous mechanistic studies have explored the variation in the expression of plant genes in response to AMF inoculation, revealing patterns of gene expression that can be utilized to determine plant’s ability to produce increased amounts of metabolites^17^. Therefore, it is important to thoroughly investigate the affinity of different AMF taxa for different plant species or cultivars in order to determine efficient AMF-host pairings for enhanced secondary metabolite synthesis. In the current work, we evaluated the interaction of two different dominant AMF species, individually and in combination with the plant host.

*Gomphrena globosa* L. is a renowned medicinal plant, indigenous to Panama, Brazil, and Guatemala and is also found in sub-tropical and tropical regions of India^18^. The flowers of this plant have medicinal properties and are used in traditional medicine for the treatment of several ailments such as hypertension, kidney problems, urinary disorders, diabetes, jaundice, whooping cough, oliguria, bronchitis and other respiratory concerns^18,19^. The medicinal benefits of *G. globosa* could be ascribed to the presence of bioactive constituents that exert physiological impact on the functioning of the human body. Prior researches have documented the existence of numerous beneficial compounds such as tocopherols, betacyanins and phenolic acids in *G. globosa*^19,20^. In a recent study, Tang et al.^18^ recorded various phenolic compounds in *G. globosa* that showcased antioxidant ability. Ferreres et al.^21^ identified 24 phenolic compounds and betacyanins from the aqueous extracts of *G. globosa* inflorescence using HPLC-DAD/ESI-MS. Betacyanins were also isolated from the *G. globosa* flowers using ion pair high-speed counter current chromatography. In-vitro scavenging micro assays revealed that *G. globosa* inflorescence has a strong ability to neutralize various reactive species and shows good antioxidant efficacy, suggesting that it may serve as a valuable source of anti-inflammatory compounds as well as antioxidants utilized in food and pharmaceutical industries^19^. Besides these beneficial effects, the impact of AMF inoculation on the secondary plant metabolite production in flowers of *G. globosa* has not been studied.

So, the primary objective of this study was to examine the effect on the secondary metabolite production and potential for antioxidant activity in the *G. globosa* flowers after inoculation with two distinct species of AMF, both individually and in combination and to select effective AMF-host combination, all of which would contribute to increased secondary metabolite production.

## Results and discussion

### AMF root colonization

**Fig. 1:**
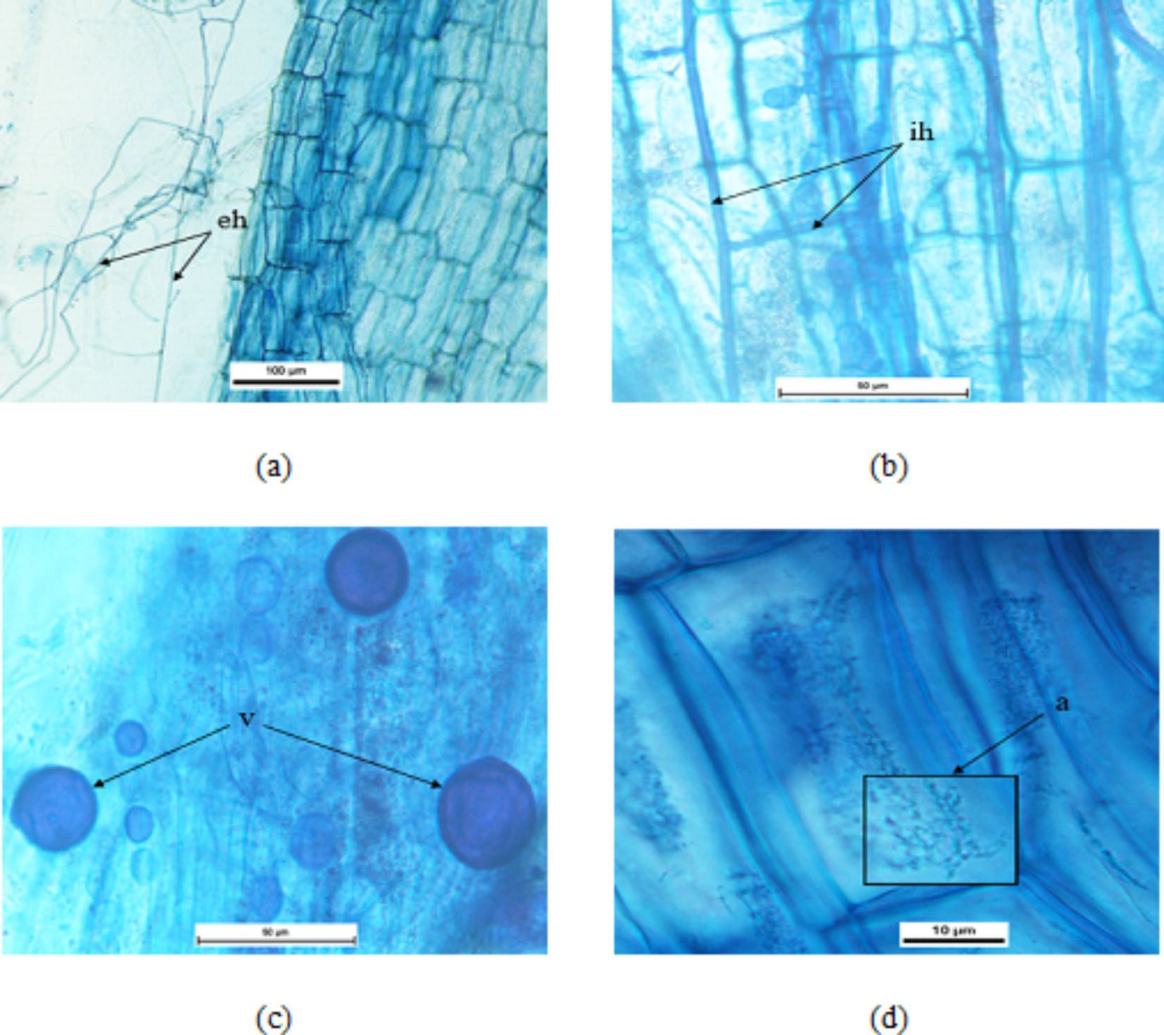
Mycorrhizal infection in the roots of *G. globosa*. Where (**a**) represents eh: extraradical hyphae, (**b**) represents ih: intraradical hyphae, (**c**) represents v: vesicles, (**d**) represents a: arbuscules.

The presence of vesicles, hyphae, and arbuscules were found in the stained roots of different AMF inoculated plants, showing effective colonization (Fig. 1). No mycorrhizal structures were detected in the *G. globosa* control roots. However, the root colonization level varied among AMF species. The highest AMF root colonization rate (66.67%) was detected in the plants exposed to combined AMF treatment, followed by plants treated with *R. intraradices* (60.00%), while the lowest colonization rate (56.67%) was observed in plants treated with *F. mosseae*. The variation in root colonization illustrates the ability of the inoculums used in this study to form mycorrhizal structures with potential effects in plants.

### AMF effect on mineral nutrient content

The concentration of macro elements including N, P, Ca, Mg and K increased significantly in the plants that were inoculated with AMF when compared with the control. Furthermore, variations were found between different AMF inoculations. Plants treated with both *F. mosseae* and *R. intraradices* exhibited significantly higher N (953.67 ± 5.69 mg/kg), P (2446.57 ± 6.08 mg/kg), Ca (8049.54 ± 7.45 mg/kg) and Mg (3440.58 ± 5.50 mg/kg) concentrations in comparison with other AMF inoculations. Non-significant differences in the K content were seen in plants inoculated with combined fungal treatment and *F. mosseae* inoculated treatment. In case of microelements, higher content of Fe (36.40 ± 0.80 mg/kg), B (87.66 ± 1.56 mg/kg), Zn (18.31 ± 0.79 mg/kg), Mo (0.43 ± 0.02 mg/kg) and Cu (4.38 ± 0.59 mg/kg) was found in combined treatment inoculated plants when compared with the control. *R. intraradices* inoculations showed significantly higher Mn content (40.60 ± 0.95 mg/kg) than plants inoculated with *F. mosseae*; however, a non-significant difference was detected in case of combined AMF treatment (Table 1).

Overall, the results demonstrated that AMF inoculation increased the nutrient absorption in *G. globosa*, which is consistent with findings of other researchers^6,22^. The observed outcome could be ascribed to the increase in the surface area, with extraradical mycelial linkage formation for the absorption. The intraradical mycelium receives nutrients from the extraradical mycelium via AMF-induced phosphate transporters in the periarbuscular membrane from where they are taken up by the host^23^. A study conducted by Javot et al.^24^, has indicated that AMF-induced phosphate transporters play an essential role in the absorption of inorganic P via mycorrhiza.

**Table 1:** Macro and micro nutrient concentration (in mg/kg) in the flowers of *G. globosa* inoculated with different treatments.

Nutrient		Treatments			
		C	Ri	Fm	Ri + Fm
Macronutrients	N	680.33 ± 4.51^a^	815.33 ± 5.03^b^	796.00 ± 6.00^c^	953.67 ± 5.69^d^
	P	1834.37 ± 5.70^a^	2333.53 ± 5.08^b^	1970.78 ± 4.75^c^	2446.57 ± 6.08^d^
	Ca	5656.93 ± 6.54^a^	7336.60 ± 6.81^b^	7140.58 ± 7.07^c^	8049.54 ± 7.45^d^
	Mg	2707.15 ± 5.01^a^	2987.76 ± 5.76^b^	2922.28 ± 5.02^c^	3440.58 ± 5.50^d^
	K	13588.10 ± 7.53^a^	13888.60 ± 7.92^b^	14923.41 ± 8.03^c^	14941.30 ± 8.11^c^
Micronutrients	Fe	28.42 ± 0.80^a^	34.69 ± 0.85^b^	31.45 ± 0.84^c^	36.40 ± 0.80^b^
	B	82.40 ± 1.35^a^	86.96 ± 1.23^b^	84.49 ± 1.67^a, b^	87.66 ± 1.56^b^
	Zn	9.57 ± 0.70^a^	16.29 ± 0.63^b^	10.21 ± 0.72^a^	18.31 ± 0.79^c^
	Cu	2.39 ± 0.46^a^	3.44 ± 0.49^a, b^	4.24 ± 0.60^b^	4.38 ± 0.59^b^
	Mo	0.31 ± 0.03^a^	0.39 ± 0.03^b^	0.40 ± 0.02^b^	0.43 ± 0.02^b^
	Mn	31.38 ± 0.85^a^	40.60 ± 0.95^b^	34.42 ± 0.97^c^	38.60 ± 0.96^b^

Values show mean ± SD (*n* = 3). Mean values with dissimilar superscript within a row are statistically significant (*p* < 0.05), deduced by Tukey’s test. Where B: boron; C: control; Ca: calcium; Cu: copper; Fe: iron; *Fm*: *F. mosseae*; K: potassium; Mg: magnesium; Mn: manganese; Mo: molybdenum; N: nitrogen; P: phosphorus; *Ri*: *R. intraradices*; Zn: zinc.

## AMF effect on extraction yield

From our study, it has been observed that different extracts have different yield extraction (Table 2). In methanol extract, the highest extraction yield (3.73%) was reported in the plant inoculated with combined treatment while the extraction yield was lowest (3.21%) in the control plants. A similar fashion was observed in chloroform extract, wherein the combined AMF inoculated plants showed the highest yield (1.15%) while the lowest (1.01%) was observed in control plants. Moreover, the findings indicate that the methanolic extract produced the highest percentage yield as compared with the chloroform extract which could be due to the high polarity of the methanol solvent. In agreement with the results of Senguttuvan et al.^25^ and Chigayo et al.^26^, who noted that the methanolic extract had the highest extraction yield in comparison to the other solvents, and could extract a wider range of plant constituents.

**Table 2:** Extractive yield (%) of methanol and chloroform extract of the flowers of *G. globosa* inoculated with different AMF treatments.

Treatments	Sample	
	Extractive yield (%)	
	ME	CE
C	3.21	1.01
*Ri*	3.45	1.09
*Fm*	3.40	1.11
*Ri* + *Fm*	3.73	1.15

Note: C represents control; CE represents chloroform extract; *Fm* represents *Funneliformis mosseae*; ME represents methanol extract; *Ri* represents *Rhizophagus intraradices*.

## Modulation in phytochemicals by AMF

**Fig. 2:**
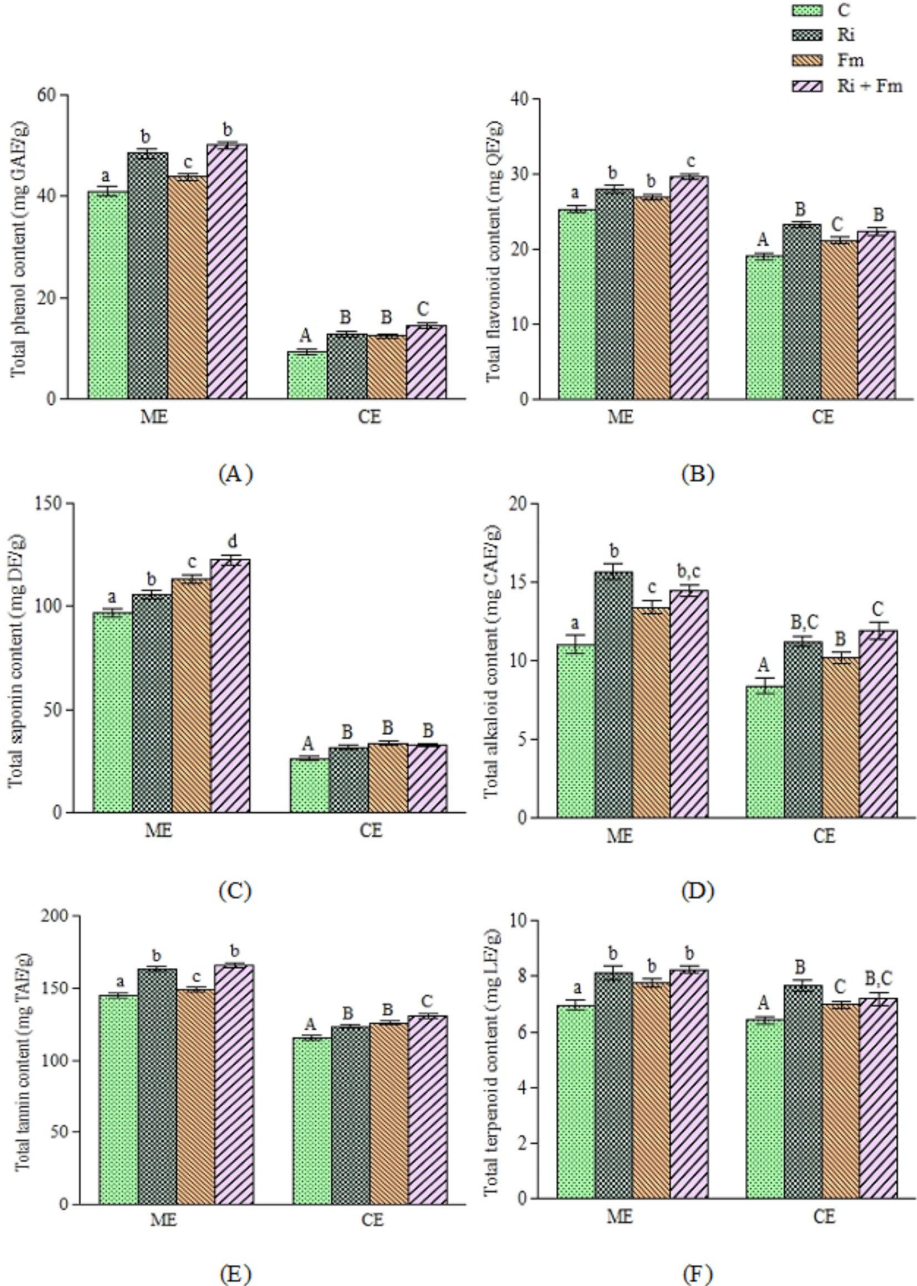
Quantitative phytochemical analysis of methanol and chloroform extract of flowers of *G. globosa* inoculated with different treatments. Where (**A**) depicts total phenol content, (**B**) total flavonoid content, (**C**) total saponin content, (**D**) total alkaloid content, (**E**) total tannin content and (**F**) total terpenoid content respectively. Different letters on bar represents significant difference at *p* < 0.05. (ME represents methanol extract and CE represents chloroform extract).

In the present study, it was found that the AMF inoculated plants showed increased production of secondary metabolites in comparison with the control (Fig. 2). In methanol extract, the maximum phenol concentration (50.11 ± 0.78 mg GAE/g) was reported in the combined AMF treatment inoculated plants which demonstrates a non-significant disparity with *R. intraradices* inoculated plants, while the lowest (41.09 ± 0.53 mg GAE/g) was observed in the control plants. In case of chloroform extract, the combination of *F. mosseae* and *R. intraradices* inoculated plants (14.59 ± 0.53 mg GAE/g) showed the maximum phenol content which was significant in comparison with the other treatments. However, no statistically significant disparity was observed in the *F. mosseae* and *R. intraradices* inoculated plants in separate treatments. AMF inoculation with combined treatment resulted in significantly higher flavonoid content (29.67 ± 0.44 mg QE/g) in the methanolic extract in comparison to other treatments. In chloroform extract, inoculation with *R. intraradices* showed the highest flavonoid content (23.27 ± 0.41 mg QE/g), which exhibits a non-significant disparity with plants inoculated with combined treatment.

Significant differences in the saponin content was found in the methanol extract when plants inoculated with different AMF were compared with non-inoculated ones. Plants inoculated with the combination of *F. mosseae* and *R. intraradices* showed significantly high concentration of saponins (122.55 ± 2.12 mg DE/g). In case of chloroform extract, the maximum saponin content was recorded in the *F. mosseae* inoculated plants which contained 28% more saponin content in comparison with the non-inoculated plants. For the alkaloid content, the maximum concentration (15.66 ± 0.50 mg CAE/g) was found in the methanolic extract of the *R. intraradices* inoculated plants, which was substantially higher than the non-inoculated plants. In case of chloroform extract, the maximum alkaloid content was observed in the combined AMF treatment (11.95 ± 0.53 mg CAE/g) which displayed a non-significant disparity in plants inoculated with *R. intraradices*. Observations of tannin content in *G. globosa* flowers revealed variations in the chloroform and methanol extracts, with combined treatment exhibiting the highest concentration (130.35 ± 1.64 mg TAE/g and 165.71 ± 1.53 mg TAE/g, respectively). In terpenoids, the combined AMF treatment inoculated plants displayed the maximum terpenoid concentration (8.24 ± 0.13 mg LE/g) in the methanol extract which demonstrated non-significant disparity with other AMF inoculated treatments. In case of chloroform extract, the maximum terpenoid content (7.65 ± 0.21 mg LE/g) was noticed in the *R. intraradices* inoculated plants, followed by combined AMF inoculated plants.

Overall, the findings demonstrated that the methanolic extract of the combined treatment inoculated plants showed a significant amount of phytochemicals in comparison with the chloroform extract. This could be attributed to the high AMF root colonization percentage in case of combined treatment inoculated plants. Moreover, *R. intraradices* was seen to exhibit greater efficiency with respect to root colonization than *F. mosseae* and hence the effect of the latter was lesser when assessed as single-species. Similar findings were observed by Ceccarelli et al.^27^ who reported that plants inoculated with *R. intraradices* exhibited higher phenolic contents compared to *F. mosseae*. According to studies by Mandal et al.^28^, Zubek et al.^29^ and Pedone-Bonfim et al.^30^, showed that colonization by AMF causes a significant increase in the plant’s nutrient uptake, particularly P, which further improves their phytochemical content. Another possible reason for the increase in metabolite levels could be linked to the biochemical changes that occur in plastids and mitochondria due to AMF, which tends to activate the tricarboxylic acid and biosynthetic plastidial pathways, resulting in the production of by-products that are used in the production of phenolic compounds^8,31^. All these explanations are possible reasons for the reported effect of the symbiotic fungi on the production of secondary metabolites in the plant, *G. globosa.*

## GC-MS study

GC-MS study was conducted to obtain a peak chromatogram of distinct phytocompounds present in methanol (Figure S1) and chloroform (Figure S2) extracts of the flower of *G. globosa* inoculated with different types of treatments. The primary active compounds identified in the methanol extract were hexadecanoic acid, methyl ester (maximum peak area of 26.40%) and 9-Octadecenoic acid (Z)-, methyl ester (maximum peak area of 23.73%) in case of combined AMF treatment. Several other compounds including methyl tetradecanoate and methyl stearate were also observed in the methanolic extract of all groups under treatments (Table 3).

**Table 3:** List of phytocompounds identified in methanol flowers extract of *G. globosa* inoculated with different treatments by GC-MS.

Compound	Mol. wt g/mol	Molecular formula	RT	Area %			
				C	Ri	Fm	Ri + Fm
Undecanoic acid, 10-methyl-, methyl ester	214	C_13_H_26_O_2_	16.91	–	1.05	0.73	1.05
Methyl tetradecanoate	242	C_15_H_30_O_2_	20.20	4.96	3.82	6.64	9.39
Hexadecanoic acid, methyl ester	270	C_17_H_34_O_2_	23.19	18.48	18.12	24.39	26.40
9-Octadecenoic acid (Z)-, methyl ester	296	C_19_H_36_O_2_	25.60	20.05	24.56	17.08	23.73
Methyl stearate	298	C_19_H_38_O_2_	25.90	10.07	15.42	13.01	10.47
Eicosanoic acid, methyl ester	326	C_21_H_42_O_2_	28.38	1.02	1.15	1.12	0.62
Methyl 11-docosenoate	352	C_23_H_44_O_2_	30.49	2.52	2.62	3.28	3.99
Docosanoic acid, methyl ester	354	C_23_H_46_O_2_	30.78	1.14	1.23	1.23	1.41

Note: C—control; *Fm*—*F. mosseae*; *Ri*—*R. intraradices*; RT—retention time.

In case of chloroform extract, hexadecane, 2,6,11,15-tetramethyl (highest peak area of 6.66%) and tetradecane, 2-methyl- (highest peak area of 6.48%) were identified as the main active constituents in the combined AMF treatment, while the lowest (2.14% and 1.65% respectively) in control plants (Table 4). Several other compounds including heptadecane and heneicosane were also identified in the chloroform extracts of all the treatments.

**Table 4:** List of phytocompounds identified in chloroform flowers extract of *G. globosa* inoculated with different treatments by GC-MS.

Compound	Mol. wt. g/mol	Molecular formula	RT	Area %			
				C	Ri	Fm	Ri + Fm
Undecane	156	C_11_H_24_	8.15	–	3.34	1.39	3.51
Dodecane	170	C_12_H_26_	9.00	–	2.68	1.07	2.89
Dodecane, 2,6,11-trimethyl-	212	C_15_H_32_	12.49	1.35	4.95	1.97	5.24
Pentadecane	212	C_15_H_32_	13.36	1.20	4.05	1.63	4.41
Hexadecane, 2,6,11,15-tetramethyl	282	C_20_H_42_	16.48	2.14	6.33	2.61	6.66
2,4-Di-tert-butylphenol	206	C_14_H_22_O	16.78	2.15	2.67	0.94	2.40
Tetradecane, 2-methyl-	212	C_15_H_32_	17.25	1.65	5.41	2.36	6.48
Heptadecane	240	C_17_H_36_	20.04	2.46	6.01	2.96	6.41
Heneicosane	296	C_21_H_44_	20.71	1.50	5.26	2.01	5.32
Phthalic acid, hept-4-yl isobutyl ester	320	C_19_H_28_O_4_	22.49	–	6.34	2.17	6.30
l-(+)-Ascorbic acid 2,6-dihexadecanoate	652	C_38_H_68_O_8_	23.74	–	4.92	1.82	5.01
Tetradecane, 2,6,10-trimethyl-	240	C_17_H_36_	23.85	3.68	4.03	1.74	4.26
Octadecane, 3-ethyl-5-(2-ethylbutyl)-	366	C_26_H_54_	26.12	–	5.05	2.43	5.41
Heptadecane, 9-hexyl-	324	C_23_H_48_	26.66	1.01	3.68	1.54	3.86
Tetrapentacontane, 1,54-dibromo-	914	C_54_H_108_Br_2_	29.21	–	2.13	0.96	2.35

Note: C—control; *Fm*—*F. mosseae*; *Ri*—*R. intraradices*; RT—retention time.

These observations demonstrated that the AMF presence increases the production of several phytochemical compounds in the flowers of *G. globosa*, as confirmed by their low presence in non-inoculated plants. Moreover, the compounds identified in both the extracts exhibit variations, signifying that the extraction of phytocompounds varies depending on the solvent used. Previous research has demonstrated that the majority of the compounds identified have a broad spectrum of pharmacological properties. 9-Octadecenoic acid (Z)-, methyl ester was found to exhibit antioxidant, anticancer, anti-inflammatory and antiandrogenic activity^32,33^ while hexadecanoic acid, methyl ester was reported to display hypocholesterolemic, anti-inflammatory, antiviral, anticancer, antioxidant, hepatoprotective and antibacterial activity^33,34^. Hexadecane, 2,6,11,15-tetramethyl was found to have antimicrobial activity^35^ whereas tetradecane, 2-methyl- was reported to exhibit antibacterial, antitumor, antifungal and cytotoxic effects^32^. The biological potential of the reported compounds in flower extract of *G. globosa* indicates the medicinal significance of the plant. The biological activity of other phytocompounds present in methanol and chloroform extracts are shown in Table 5.

**Table 5:** Phytocompounds detected in the methanol extract and chloroform extract of flower through GC-MS analysis, along with the compound nature and their biological activities.

Compound	Chemical nature	Biological activity	References
2,4-Di-tert-butylphenol	Phenol	Antimicrobial, antioxidant, antidiabetic, and anticancer	^36,37^
9-Octadecenoic acid (Z)-, methyl ester	Methyl ester of unsaturated fatty acid	Anti-inflammatory, anemiagenic, antioxidant, dermatitigenic, insectifuge, antiandrogenic, anticancer, 5-alpha reductase inhibitor and hypocholesterolemic	^32,33,38^
Docosanoic acid, methyl ester	Fatty acid	Diagnostic and therapeutic	^39^
Dodecane	Alkane	Food additives, lubricants, flavoring agents, antibacterial and activator of heat shock response (HSR) signaling pathway activators	^32,40^
Dodecane, 2,6,11-trimethyl-	Aliphatic alkane	Antimicrobial	^41^
Eicosanoic acid, methyl ester	Saturated fatty acid	Inhibitors of α-glucosidase	39
Heneicosane	Alkane	Antiasthmatic, urine acidifiers and antimicrobial	^34,42^
Heptadecane	Alkane hydrocarbon	Antimicrobial	^34^
Heptadecane, 9-hexyl-	Hydrocarbon	Antifungal	^43^
Hexadecane, 2,6,11,15-tetramethyl	Hydrocarbon	Antimicrobial	^35^
Hexadecanoic acid, methyl ester	Fatty acid methyl ester	Anticancer, antiviral, anti-inflammatory, alpha reductase inhibitor, hypocholesterolemic, anticoronary, antioxidant, antibacterial, insectifuge, nematicide, antihistaminic, antiacne, antiandrogenic, antiarthritic, lipoxygenase inhibitor, antieczemic and hepatoprotective	^33,34,44^
l-(+)-Ascorbic acid 2,6-dihexadecanoate	Reductone	Protects LDL from peroxidation, antitumor, antibacterial, anti-inflammatory, antioxidant and decreases the progression of atherosclerosis	^45,46^
Methyl stearate	Fatty acid methyl ester	Regulate lipid metabolism in intestine, anti-inflammatory, anti-diarrheal, antinociceptive, anti-proliferative, nematicidal, cytotoxic, antioxidant and antifungal	^44,47^
Methyl tetradecanoate	Fatty acid methyl ester	Antimicrobial	^44^
Octadecane, 3-ethyl-5-(2-ethylbutyl)-	Alkane	Antimicrobial	^48^
Pentadecane	Alkane	Antioxidant and antimicrobial	^34,41^
Phthalic acid, hept-4-yl isobutyl ester	Aromatic compound	Antioxidant and anti-inflammatory	^49^
Tetradecane, 2,6,10-trimethyl-	Saturated isoprenoid hydrocarbon	Antiasthmatic and anti-inflammatory	^50^
Tetradecane, 2-methyl-	Alkane	Antitumor, antimicrobial and cytotoxic	^32^
Tetrapentacontane, 1,54-dibromo-	Aliphatic hydrocarbon	Antioxidant	^51^
Undecane	Alkane	Antimicrobial, carcinogens and enzyme inhibitors	^38^

## HPTLC study

In order to attain optimal resolution and consistent peaks, different solvent compositions were investigated. HPTLC chemical profiling of methanol and chloroform extracts revealed distinct bands that were separated differently on the chromatographic plate when seen under UV light (254 nm and 366 nm). Each band indicates distinct phytocompounds present in the extract. Standard spots were easily seen and contrasted with the sample ones. The presence of kaempferol and benzoic acid was observed in both the extracts. The results, as illustrated in Figure S3, shows that the methanol extract had more bands and displayed better separation than the chloroform extract.

Furthermore, the identification of the bands of kaempferol and benzoic acid in both the extracts were validated by comparing their absorption spectra with those of standards using a CAMAG Scanner. The separation of compounds was seen in different treatments with chromatogram peaks in methanol extract and chloroform extract when scanned with mobile phase of flavonoids (Figure S4) and phenols (Figure S5). The standard displayed fairly good separation in the selected solvent systems, with kaempferol having maximum Rf value of 0.85 and benzoic acid with maximum Rf value of 0.97, as displayed in Figure S6. The band region with maximal Rf value ranging between 0.76 and 0.79 corresponds to kaempferol and Rf value between 0.94 and 1.01 corresponds to benzoic acid in case of the test solution, as observed in methanol as well as chloroform extract with varied treatments. Furthermore, as indicated in Table 6, the amount of kaempferol and benzoic acid was measured in both the extracts. Arthi and Prasanna^52^ reported that HPTLC analysis of *G. globosa* flowers showed the presence of quercetin with toluene: ethyl acetate: formic acid (5:4:1) as the mobile phase. Overall, our findings showed that the highest amount of kaempferol and benzoic acid was detected in the methanolic extract of combined AMF treatment.

**Table 6:** HPTLC profile for standard (kaempferol and benzoic acid) and *G. globosa* flower extracts (methanol and chloroform) with different treatments.

Standard and treatments		Maximum Rf	Area	Amount (%)	Substance assigned
Standard		0.85	16915.9	–	Kaempferol
Methanol extract	C	0.77	2493.3	0.40	
	*Ri*	0.76	3149.1	0.49	
	*Fm*	0.76	2833.3	0.45	
	*Ri* + *Fm*	0.76	6527.6	0.90	
Chloroform extract	C	0.78	628.5	0.18	
	*Ri*	0.77	3525.7	0.53	
	*Fm*	0.79	4702.7	0.68	
	*Ri* + *Fm*	0.77	5672.9	0.79	
Standard		0.97	6006.8	–	Benzoic acid
Methanol extract	C	0.95	2748.3	1.97	
	*Ri*	0.95	4365.2	3.75	
	*Fm*	0.96	3645.8	2.96	
	*Ri* + *Fm*	0.94	6260.3	5.83	
Chloroform extract	C	1.01	4111	3.47	
	*Ri*	0.94	4789.7	4.22	
	*Fm*	0.97	2786.6	2.01	
	*Ri* + *Fm*	0.95	10574.2	10.57	

Note: C—control; *Fm*—*F. mosseae*; *Ri*—*R. intraradices*.

### FT-IR study

**Fig. 3:**
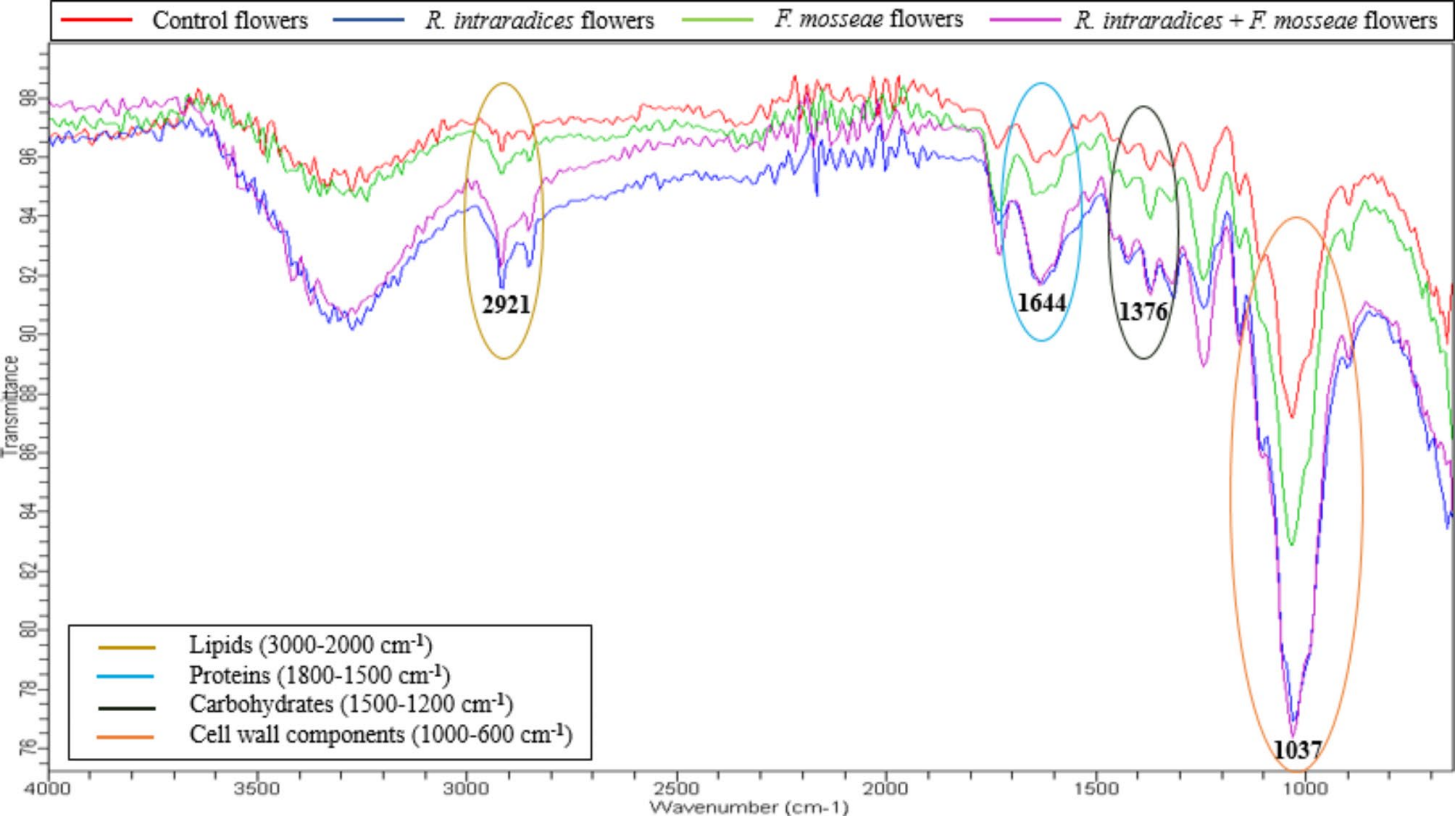
FT-IR spectra of *G. globosa* flowers inoculated with different treatments.

FTIR spectroscopy is very sensitive and reliable technique for identifying bioactive compounds. In the present study, this technique was used to determine the functional groups of bioactive components found in the *G. globosa* flowers inoculated with various AMF treatments as demonstrated by differences in the peaks (Fig. 3). The flower samples were scanned from 650 to 4000 cm^−1^ wave number range and the regions were assigned as lipids from 3000–2000 cm^−1^, proteins from 1800–1500 cm^−1^, carbohydrates from 1500–1200 cm^−1^ and cell wall components from 1000–600 cm^−1^ wave number. Table S1 displays the FT-IR spectral data peaks of each treatment, along with their probable functional groups. The existence of alcohols, aldehydes, alkanes, alkenes, amines, esters, carboxylic acids and nitro compounds was observed in all the treatments and it is noteworthy that these groups form essential components of various secondary plant metabolites like polyphenol, tannins, flavonoids, terpenoids and alkaloids. However, high intensity of the peaks was observed with the combined treatment inoculated plants indicating maximum accumulation of metabolites whereas the lowest was observed in the control plants. Crisan et al.^53^ found that the increase in the concentration of phytochemicals could be due to the improvement in nutrition.

## AMF effect on the antioxidant activity

Antioxidants are the substances that interact with and counteract free radicals, thereby inhibiting their ability to harm biological molecules^54^. They are also known as “free radical scavengers.” In the current study, three distinct assays were employed to assess the antioxidant activity. These included the i.e. DPPH assay, ABTS assay and FRAP assay. It was also observed that all the extracts demonstrated a dose-dependent antioxidant activity. The DPPH, a stable free radical, is widely used to assess the antioxidant ability to scavenge free radicals. Antioxidant compounds have the ability to quench DPPH and convert it into colorless products^55^. According to our findings, the highest antioxidant activities were noticed in the AMF inoculated plants when compared with the control plants. In DPPH assay, the methanol extract of combined treatment showed the highest antioxidant potential with a very low IC_50_ value of 401.39 ± 1.96 µg/mL as compared to the control plants having IC_50_ value of 515.32 ± 2.59 µg/mL. In case of chloroform extract, the *R. intraradices* inoculated plants exhibited the highest antioxidant activity (IC_50_ value 536.12 ± 2.41 µg/mL), followed by plants with combined treatment (IC_50_ value 539.77 ± 2.42), while the lowest antioxidant activity (IC_50_ value 606.29 ± 2.61 µg/mL) was reported in the non-inoculated plants. Moreover, for ascorbic acid, an IC_50_ value of 7.27 ± 0.25 µg/mL was observed and is shown in Table 7.

**Table 7:** Antioxidant activity of *G. globosa* flower extracts inoculated with different treatments by DPPH, ABTS (IC_50_ expressed in µg/ml) and FRAP [expressed in terms of µM fe (II) equivalent] methods.

Standard	DPPH		ABTS		FRAP	
Sample Treatments	ME	CE	ME	CE	ME	CE
C	515.32 ± 2.59^a^	606.29 ± 2.61^a^	103.37 ± 2.24^a^	179.61 ± 1.97^a^	5631.71 ± 6.51^a^	2988.76 ± 4.52^a^
*Ri*	487.52 ± 2.32^b^	536.12 ± 2.41^b^	87.19 ± 2.45^b^	143.08 ± 1.63^b^	7632.38 ± 7.50^b^	3417.95 ± 5.00^b^
*Fm*	501.47 ± 2.41^c^	571.72 ± 2.57^c^	97.45 ± 1.60^c^	152.03 ± 2.20^c^	6488.09 ± 7.57^c^	3310.70 ± 4.70^c^
*Ri* + *Fm*	401.39 ± 1.96^d^	539.77 ± 2.42^b^	71.18 ± 2.54^d^	119.01 ± 2.46^d^	8774.73 ± 7.60^d^	4202.52 ± 5.08^d^
Ascorbic acid (Standard)	7.27 ± 0.25		2.93 ± 0.02		5524.33 ± 6.03	

Values show mean ± SD (*n* = 3). Mean values with dissimilar superscript within a column are statistically significant (*p* < 0.05), as analyzed by Tukey’s test. Where C represents control; CE represents chloroform extract; *Fm* represents *F. mosseae*; ME represents methanol extract; *Ri* represents *R. intraradices*.

Likewise, in ABTS assay, the inclusion of hydrogen-donating antioxidant molecules decreases the production of ABTS radical cation and results in the decolorization of ABTS^56^. Highest ABTS radical scavenging capabilities were observed in combined AMF treatment inoculated plants, with IC_50_ value 71.18 ± 2.54 µg/mL and 119.01 ± 2.46 µg/mL in case of methanol and chloroform extract respectively, whereas the control plants exhibited the IC_50_ value of 103.37 ± 2.24 µg/mL and 179.61 ± 1.97 µg/mL in the same mentioned extracts. The standard ascorbic acid showed IC_50_ value of 2.93 ± 0.02 µg/mL as shown in Table 7.

The FRAP assay is predicated on the reduction of a colorless ferric-tripyridyltriazine complex to blue colored ferrous-tripyridyltriazine at a low pH by the action of antioxidants that donate electrons^57^. The FRAP activity of the extracts increases with the concentration. Significant differences in the antioxidant activity were found in the methanol extract and chloroform extract when plants inoculated with different AMF treatments were compared with non-inoculated plants. In methanol extract, the combined treatment inoculated plants exhibited a substantially higher reducing power activity i.e. 8774.73 ± 7.60 µM Fe (II) equivalent. Instead, a lower activity was found in the control plants i.e. 5631.71 ± 6.51 µM Fe (II) equivalent. In chloroform extract, the combined *F. mosseae* and *R. intraradices* treatment inoculated plants showed the maximum reducing power activity of 4202.52 ± 5.08 µM Fe (II) equivalent while the lowest activity of 2988.76 ± 4.52 µM Fe (II) equivalent was detected in the control plants. For ascorbic acid, the activity of 5524.33 ± 6.03 µM Fe (II) equivalent was observed, as shown in Table 7.

The overall results demonstrated that the methanolic extract of the combined treatment seemed to have the maximum antioxidant potential. This could be attributed to the increased levels of flavonoid and phenol in the plant extract. Numerous studies have shown that mycorrhizal fungi effectively increase the accumulation of flavonoids and phenols because they contain hydroxyls, which have redox characteristics and cause the radical scavenging effect^55,58^. Similar findings were made by Avio et al.^59^, who found an increased antioxidant activity in the *Lactuca sativa* leaves after inoculation with mycorrhiza. Rashidi et al.^60^, observed high concentrations of phenol based compounds and antioxidant activities in the reproductive organs and roots of *Ipomoea purpurea*, *Solanum nigrum* and *Digitaria sanguinalis* inoculated with AMF. Several reports stated that the increased antioxidant activity in the extracts could be due to the synthesis of phenolic compounds in plants inoculated with AMF which provides defense against both biotic and abiotic stresses^61^. Thus, according to the findings, it is suggested that inoculation of both *F. mosseae* and *R. intraradices* in *G. globosa* could potentially serve as a source of antioxidants for pharmaceutical formulations.

## Conclusion

The findings showed that the co-inoculation of *R. intraradices* and *F. mosseae* increased the secondary metabolites synthesis in the flowers of *G. globosa*. This increase is due to the successful host colonization and the level of cellular interactions that facilitate improved acquisition and transfer of nutrients at the interface between the AMF and the host. Furthermore, AMF inoculation improved the antioxidant activity, which is ascribed to the higher concentrations of bioactive compounds including phenols and flavonoids, which function as free radical scavengers. Thus, this study on *G. globosa* could serve as a basis for future pharmacological and drug design research. Moreover, additional research is needed to determine the potential mechanism of action triggered by AMF in boosting the examined phytochemicals.

## Materials and methods

### AMF inoculum

The AMF inoculum of *Funneliformis mosseae* and *Rhizophagus intraradices* was obtained from the University of Agricultural Sciences in Bangalore, India. The inoculums were mass multiplied in a sterilized mixture of sand and soil (3:1) using the host plant, *Sorghum vulgare*.

### Pot experiment design

The seeds of *G. globosa* were acquired from the Shoolini University, Solan, Himachal Pradesh, India. Before germinating the seeds in petri plates, they were surface sterilized with 0.1% (w/v) mercuric chloride. After a week, healthy seedlings were transplanted in separate pots filled with sterilized sand and soil (3:1) mixture. A completely randomized design was used for the experiment with following treatment levels: (1) control: plants that were not treated with any AMF; (2) AMF treated: plants that were treated with either *R. intraradices* or *F. mosseae* alone or with a combination of both. In each AMF treated plants, 10% of the multiplied inoculum (containing colonized root segments, fungal spores and rhizospheric soil) was added^62^. A total of thirty replicates were used for each AMF species tested as well as for the control. The plants were watered regularly and maintained within the greenhouse. In every 15 days of seedling growth, 50 mL of the Hoagland medium solution (without KH_2_PO_4_) was added. The harvesting of the plants was done after a growth period of four months. The roots of each treatment were delicately cleared of soil particles, thoroughly rinsed with water and thereafter stained in order to visualize the mycorrhizal infection.

### Microscopic examination of AMF infection in roots

The “rapid clearing and staining method” was used to visualize the infection^63^. The roots segments were kept in 10% KOH (w/v) solution for a duration of 30 min at 90 °C before being washed with distilled water. Afterwards, the root samples were treated in 1% HCl (v/v) for 20 min and then stained for 15–30 min in 0.05% (w/v) trypan blue in lactophenol. The root segments were randomly selected from the stained samples to assess the presence of AMF structures using a light microscope. The following formula was used for calculating the percentage of AMF colonization:

$${\text{Root colonization }}(\%)=\frac{{\text{Number of infected root segments}}}{{\text{Number of root segments observed}}} \times 100$$

### Determination of mineral nutrients

For determination of total nitrogen (N), a Kel Plus nitrogen estimation system was used. 0.5 g of powdered flower sample was digested with sulphuric acid and then distill with sodium hydroxide. After distillation, the distillate obtained from the Kel Plus distillation unit was subjected to titration using 0.1 (N) H_2_SO_4_^64^.

For determination of phosphorus (P), calcium (Ca), magnesium (Mg), potassium (K), iron (Fe), boron (B), zinc (Zn), copper (Cu), molybdenum (Mo) and manganese (Mn), 0.3 g of samples were microwave digested using 5 mL of nitric acid and 1 mL of hydrogen peroxide. The solution was then filtered and diluted with distilled water, prior to its analysis using an inductively coupled plasma-optical emission spectrometer (ICP-OES)^65^.

### Extract preparation

The flowers of *G. globosa* from each treatment was taken for the current study. The samples were thoroughly cleaned with distilled water and were air-dried for 10–15 days at room temperature. The desiccated samples were then pulverized to prepare a fine powder using a mixer grinder. For extraction, 10 g of powdered flowers were mixed with 100 mL chloroform and 100 mL methanol separately and was kept in a rotary orbital shaker (120 rpm speed) for 72 h. After mixing the two solvents, the extracts were filtered and then dried at 37 ºC in a hot air oven before being placed in the refrigerator at 4 ºC for subsequent analysis^66^.

### Extraction yield

The percentage yield for individual extract was determined^67^ using the following formula:

$${\text{Extraction yield }} (\%)=\frac{{\text{Weight of the crude extract}}}{{\text{Weight of the powdered sample}}} \times 100$$

### Phytochemical analysis

#### Assessment of total phenols

The Folin-Ciocalteu technique^68^ was utilized for assessment of total phenolic content of the extracts. 1 mL of extract (1 mg/mL concentration) was mixed with 5 mL of 10% Folin-Ciocalteu reagent and 4 mL of 7.5% sodium carbonate solution. After 2 h of incubation at room temperature, the absorbance at 765 nm was measured using a UV/Visible spectrophotometer. A calibration curve using gallic acid (5–60 µg/mL) as a reference was plotted and quantification of the total phenol content was done and displayed as gallic acid equivalents (GAE).

#### Assessment of total flavonoids

The aluminum chloride colorimetric technique^69^ was used for assessing the total flavonoid content. 0.5 mL of extract (1 mg/mL concentration) was subjected to treatment with 1.5 mL of 95% ethanol, 0.1 mL of 10% aluminum chloride solution and 0.1 mL of 1 M potassium acetate. Distilled water was added to make the final volume 5 mL. After 30 min of incubation at room temperature, the absorbance at 415 nm was measured. A calibration curve using quercetin (5–60 µg/mL) was plotted and the total flavonoid content was measured and displayed as quercetin equivalents (QE).

#### Assessment of total saponins

The vanillin-sulphuric acid colorimetric technique^70^ was used for assessing the total saponin content. 0.5 mL of extract (1 mg/mL concentration) was subjected to treatment with 0.5 mL of 8% vanillin and 5 mL of 72% concentrated sulfuric acid. The mixture was heated in water bath at 60 ºC for 10 min and later placed for 5 min in an ice-water. After cooling, the absorbance at 560 nm was recorded. A calibration curve using diosgenin (ranging between 5 and 60 µg/mL) was plotted and the total saponin content was measured and displayed as diosgenin equivalents (DE).

#### Assessment of total alkaloids

The bromocresol green technique^71^ was utilized for assessing the total alkaloid content. 1 mL of extract (1 mg/mL prepared in 2 N HCl) was subjected to treatment with 5 mL each of the phosphate buffer with pH = 4.7, bromocresol green solution and chloroform. The mixture was shaken vigorously so that the chloroform layer gets separated and was collected in a conical flask, with further addition of chloroform to make the final volume to 10 mL. The absorbance at 470 nm was recorded and a calibration curve using caffeine (5–60 µg/mL) was plotted. The content for total alkaloids was measured and displayed as caffeine equivalents (CAE).

#### Assessment of total tannins

The method reported by Morsy^72^ was used to assess the total tannin content. 0.5 mL of extract (1 mg/mL concentration) was subjected to treatment with 1 mL of 1% potassium ferricyanide and 1 mL of 1% ferric chloride. The final volume was made 10 mL by addition of distilled water. The absorbance at 720 nm was recorded and a calibration curve using tannic acid (ranging between 5 and 60 µg/mL) was plotted. The total tannin content was measured and displayed as tannic acid equivalents (TAE).

#### Assessment of total terpenoids

The total terpenoid content was assessed using the method reported by Truong et al.^73^. 1 mL of extract (1 mg/mL) was subjected to treatment with 2 mL of chloroform and 200 µL of concentrated sulfuric acid. The mixture was then kept in the dark at room temperature. A reddish-brown precipitate was formed after 2 h. A volume of 3 mL of 95% methanol was introduced into the reaction mixture after the supernatant was decanted. The absorbance at 538 nm was recorded and a calibration curve using linalool (ranging between 5 and 25 µg/mL) was plotted. The total terpenoid content was measured and displayed as linalool equivalents (LE).

### Gas chromatography-mass spectroscopy (GC-MS) study

The analysis of phytochemicals extracted from *G. globosa* flowers with different types of treatments was carried out using triple-quadrupole GCMS-MS spectrometer (Thermofisher) equipped with TG-5MS capillary column (40 m length × 0.15 mm diameter × 0.15 μm thickness). The carrier gas employed was pure helium gas (99.99% purity) with a flow rate of 0.7 mL/min. A splitless mode was used with an injection volume of 1 µL, while the injector temperature was maintained at 270 °C. The column oven temperature was adjusted to 70 °C with initial hold time of 1 min and equilibration period of 2 min. The temperature was gradually increased to 270 °C at ramp rate of 7 °C/min and a holding duration of 20 min. The total run time was 50 min^42,65^. The data was analyzed using a total ion chromatogram to identify and quantify the compounds. The compounds were recognized by comparing their retention time and mass spectrum patterns with those held in the National Institute of Standards and Technology library.

### High performance thin layer chromatography (HPTLC) study

Chromatographic grade methanol was used to prepare 1 mg/mL standard solutions (kaempferol and benzoic acid) and 20 mg/mL flower extracts (chloroform and methanol) with different treatments. Using CAMAG Linomat 5 sample applicator, the samples were loaded over HPTLC plates coated with silica gel 60 F 254. The plates were thereafter kept inside a TLC twin trough chamber and run with their appropriate mobile phases. The solvent systems containing toluene, ethyl acetate and glacial acetic acid in 30:40:4 ratio (for kaempferol) and toluene, acetone, and formic acid in 4.5:4.5:1 ratio (for benzoic acid) presented the most effective outcomes^74^. The plates were further dried and placed inside a photo documentation chamber i.e. CAMAG TLC scanner, where the recording of images was done under UV light at wavelengths of 254 nm and 366 nm. Additionally, the chromatographic retardation factor values were also measured.

### Fourier transform infrared spectroscopy (FTIR) study

The FTIR analysis of flower samples was carried out to study the functional groups. A minute quantity of the dried powder sample was placed onto the sample holder under a consistent pressure. The IR peak absorbance was detected in the region of 4000 cm^−1^ to 650 cm^−1^ with a spectral resolution of 4 cm^−1^ using an Agilent Cary 630-FTIR spectrometer. The identification of functional groups was done by comparison of the absorption frequencies with those documented in the literature as referenced from Nikalje et al.^75^.

### Determination of antioxidant activity

#### 2,2-diphenyl-1-picrylhydrazyl (DPPH) assay

3 mL of extract at different concentrations of 30, 60, 120, 240, 480, and 960 µg/mL was treated with 1 mL of a 0.1 mM DPPH solution. The mixture was kept at room temperature in dark for 30 min. Similarly, different ascorbic acid concentrations of 0.5, 1, 2, 4, 8, and 16 µg/mL were prepared. As a control, the DPPH solution prepared in methanol was utilized and the absorbance at 517 nm was recorded^76^. The inhibition % was computed as:

$${\text{DPPH inhibition }}\%= \left(\frac{{{\text{A}}}_{{\text{C}}}-{{\text{A}}}_{{\text{E}}}}{{{\text{A}}}_{{\text{C}}}}\right)\times 100$$

where A_C_ and A_E_ is the absorbance value for control and plant extracts, respectively.

#### 2,2-azinobis-(3-ethylbenzothiazoline-6-sulfonic acid) (ABTS) assay

The working ABTS^•+^ was produced by mixing 7 mM ABTS solution and 140 mM potassium persulphate solution in a 5:0.08 (v/v) ratio in distilled water and incubating it in dark for 14 h at room temperature. A blue/green colour ABTS^•+^ chromophore was formed whose absorbance was adjusted to 0.700 (± 0.020) by diluting it with ethanol. After that, 1 mL of prepared ABTS^•+^ was treated with 2 mL of varying concentrations of extract (30, 60, 120, 240, 480 and 960 µg/mL) and ascorbic acid (0.5, 1, 2, 4, 8, 16 µg/mL), separately. The samples were vortexed and the absorbance at 734 nm was recorded^65^. The inhibition % was computed as:

$${\text{ABTS inhibition }} (\%)=\left(\frac{{{\text{A}}}_{{\text{C}}}-{{\text{A}}}_{{\text{E}}}}{{{\text{A}}}_{{\text{C}}}}\right)\times 100$$

where A_C_ and A_E_ is the absorbance value of the control and plant extracts, respectively.

#### Ferric-reducing antioxidant power (FRAP) assay

The working FRAP reagent was produced by mixing 300 mM sodium acetate buffer (pH = 3.6), 10 mM tripyridyltriazine solution in 40 mM HCl solution and 20 mM ferric chloride solution in a ratio 10:1:1 (v/v/v) and incubating it in dark for 30 min. 2.9 mL of FRAP reagent was treated with 0.1 mL of different concentrations of extract and FeSO_4_ (1, 150, 300, 600, 1200, 2400 µM) and ascorbic acid (30, 60, 120, 240, 480, 960 µg/mL), separately. The prepared samples were agitated and placed in dark for 30 min at 37 ºC. An equal amount of DMSO (0.1 mL) was used as a control and the absorbance at 593 nm was recorded^77^. Here, FeSO_4_ was used for calibration, and the results were compared with positive control, ascorbic acid. The FRAP activity measurement was carried out, displayed as ferrous equivalent (FE) and expressed in µM.

### Statistical analysis

The results were subjected to analysis of variance (ANOVA) using software Graph Pad Prism. The significance level was set at *p* < 0.05 and the Tukey test was used to separate the means in order to find if the values were substantially different. The experiments were carried out in triplicates and the findings were represented as mean values ± standard deviation (SD).

## Electronic supplementary material

Below is the link to the electronic supplementary material.

Supplementary Material 1

## Data Availability

Data will be made available on request from the corresponding author end.
