# Supplementary material for: Assessing the potential role of arbuscular mycorrhizal fungi in improving the phytochemical content and antioxidant properties in Gomphrena globosa
Source: Sci Rep. 2024 Oct 1;14:22830. doi: 10.1038/s41598-024-73479-5 (PMC11445267; doi:10.1038/s41598-024-73479-5)
Supplement: Supplementary file 1 — Supplementary Material 1 [file 41598_2024_73479_MOESM1_ESM.docx]

**Table S1.** FT-IR spectra illustrating the identified peaks and possible functional groups in the flowers of *G. globosa*.

| **Wave number (cm^-1^)** | | | | **Probable functional groups** |
| --- | --- | --- | --- | --- |
| **C** | ***Ri*** | ***Fm*** | ***Ri* + *Fm*** |  |
| Lipids (3000-2000 cm^-1^) | | | | |
| 2921 | 2921 | 2921 | 2921 | S, C—H stretch (Alkenes), O—H stretch (Carboxylic acids), |
| - | - | - | 2895 | S, O—H stretch (Carboxylic acids), (CO)—H stretch (Aldehydes), C—H stretch (Alkanes) |
| Proteins (1800-1500 cm^-1^) | | | | |
| - | 1734 | 1737 | 1737 | C꞊C (benzenes), C꞊O (Esters, aldehydes ketones, carboxylic acids) |
| 1644 | 1637 | 1652 | 1633 | C꞊C (Alkenes, benzenes) (Proline), C꞊O stretch (Ketones, carboxylic acids), C—O stretch (Amides), N—H bend (Amines, nitro compounds) |
| Carbohydrates (1500-1200 cm^-1^) | | | | |
| 1376 | 1376 | 1376 | 1372 | O—H bend (Alcohols), CO—H bend (Aldehydes), N꞊O stretch (Nitro compounds), C—O stretch (Esters) |
| 1320 | 1320 | 1324 | 1320 | O—H bend (Alcohols, carboxylic acids), S(꞊O)2 stretch (Sulfones), N꞊O stretch (Nitro compounds) |
| 1249 | 1246 | 1246 | 1246 | C—O stretch (Alcohols, esters, ethers), C—N stretch (Amines), O—H bend (Carboxylic acids) |
| Cell wall components (1000-600 cm^-1^) | | | | |
| - | - | 825 | - | C—C (Chlorides), ꞊C—H bend (Alkenes, benzene), C—N stretch (Amines) |
| - | - | - | 765 | C—C stretch, ꞊C—H bend (Benzene), C—N stretch (Amines) |
| 665 | - | - | 665 | C—C stretch (Chlorides), ꞊C—H bend (Benzene), C—N stretch (Amines) |

Note: C: control; *Fm*: *F. mosseae*; *Ri*: *R. intraradices.*

| 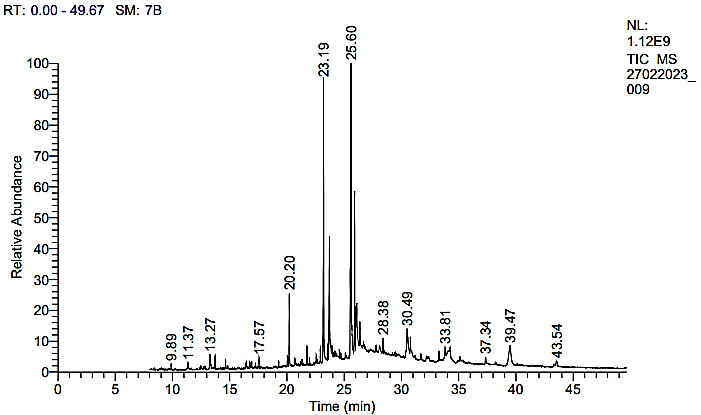 | 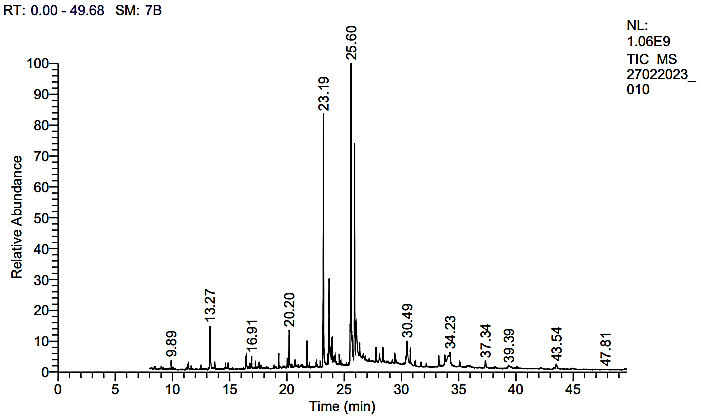 |
| --- | --- |
| **(a)** | **(b)** |
| 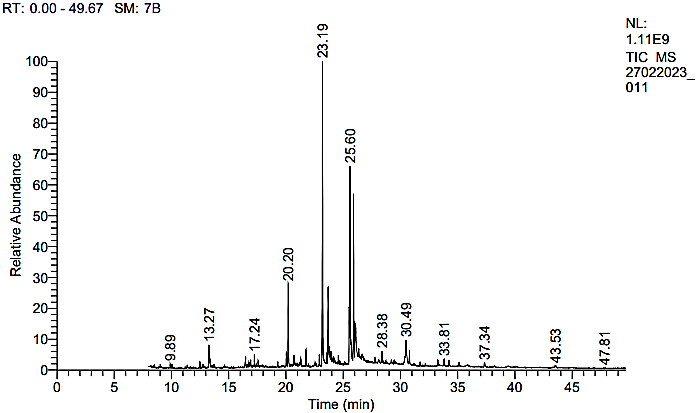 | 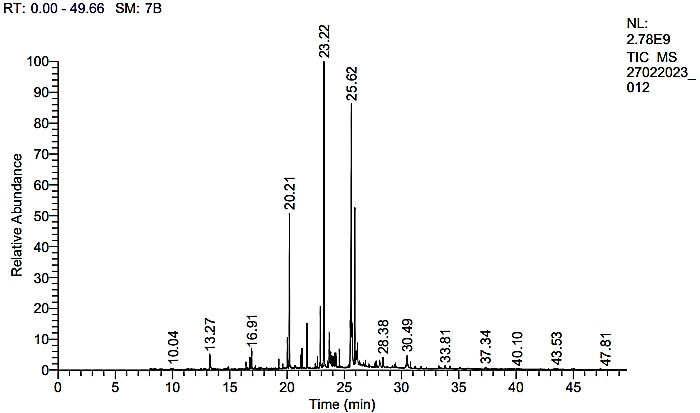 |
| **(c)** | **(d)** |

**Figure S1.** GC-MS chromatogram of methanol flower extract of *G. globosa* inoculated with different treatments (a) Control (b) *R. intraradices* (c) *F. mosseae* (d) *R. intraradices* + *F. mosseae.*

| **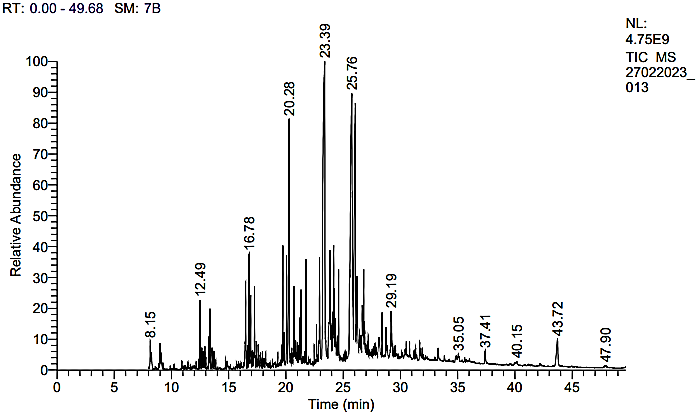** | 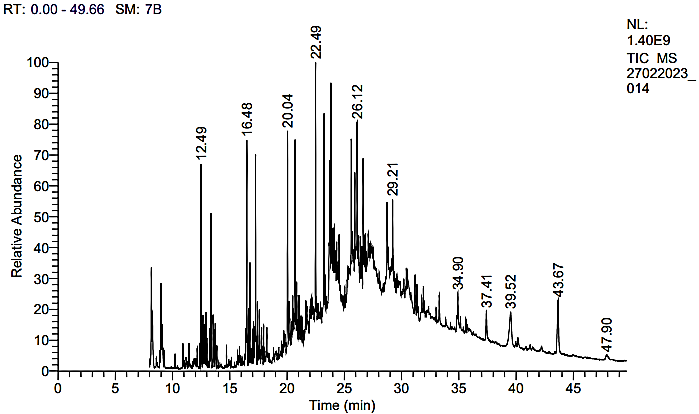 |
| --- | --- |
| **(a)** | **(b)** |
| 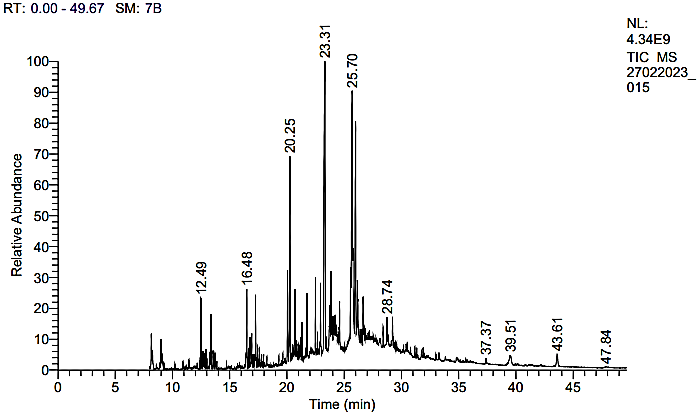 | 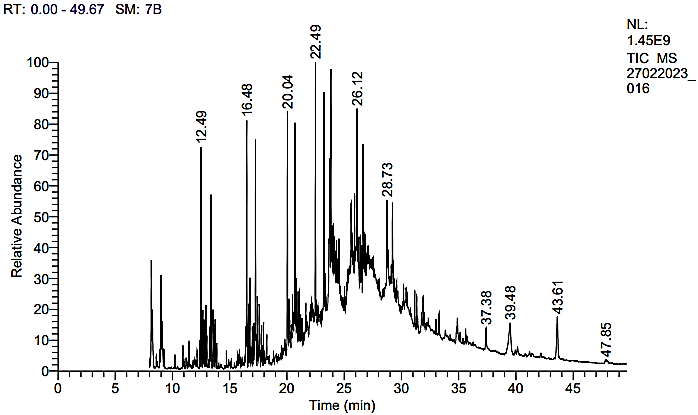 |
| **(c)** | **(d)** |

**Figure S2.** GC-MS chromatogram of chloroform flowers extract of *G. globosa* inoculated with different treatments (a) Control (b) *R. intraradices* (c) *F. mosseae* (d) *R. intraradices* + *F. mosseae.*

| 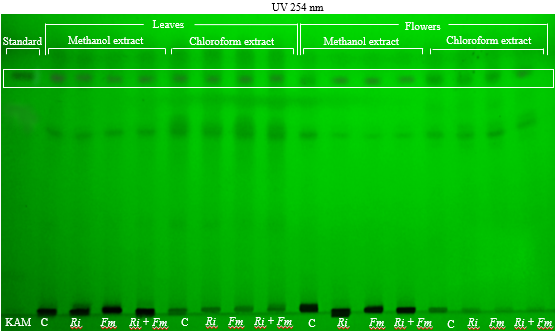 | 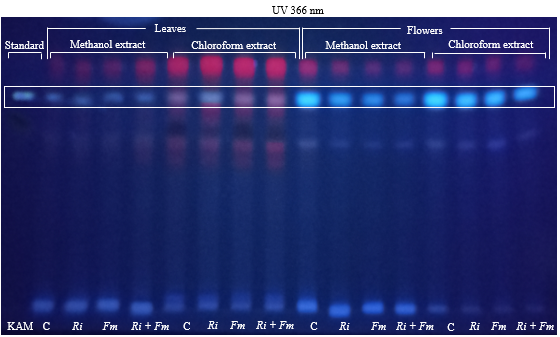 |
| --- | --- |
| (a) | |
| 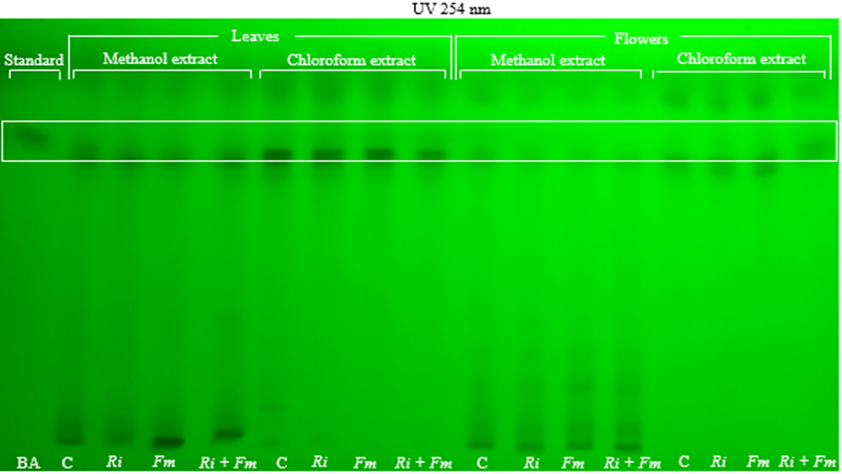 | 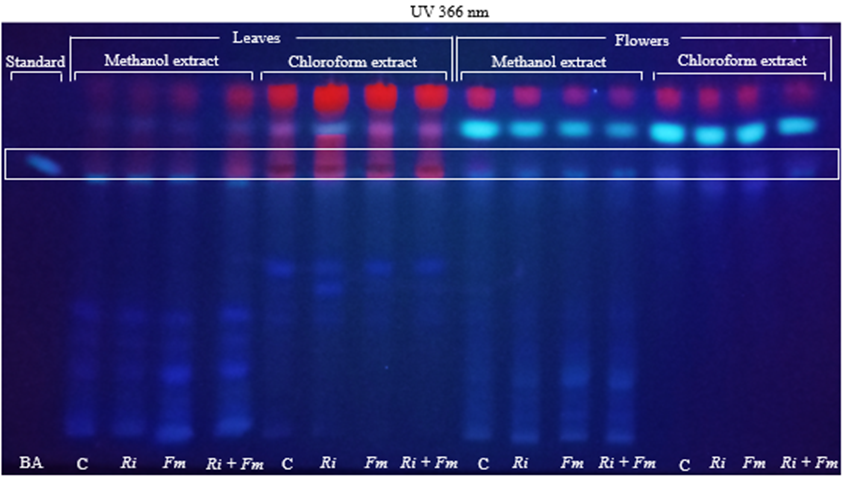 |
| (b) | |

**Figure S3.** HPTLC band chromatogram using solvent system (a) toluene-ethyl acetate-glacial acetic acid (30:40:4) solvent system for flavonoids, (b) acetone-toluene-formic acid (4.5: 4.5: 1) solvent system for phenols under UV 254 nm and UV 366 nm.

| 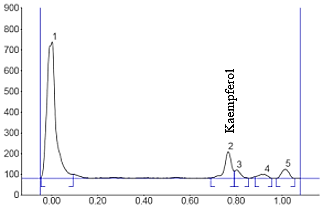 | 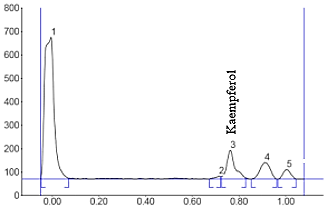 |
| --- | --- |
| (a) | (b) |
| 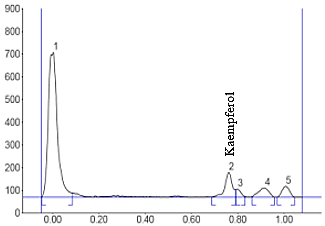 | 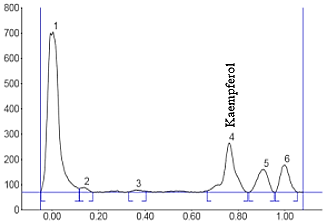 |
| (c) | (d) |
| 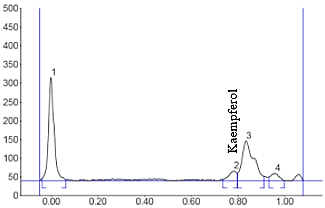 | 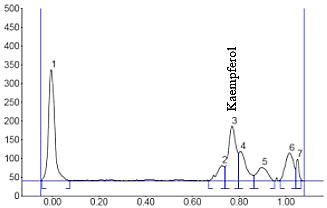 |
| (e) | (f) |
| 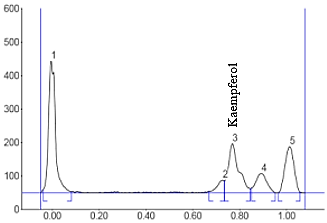 | 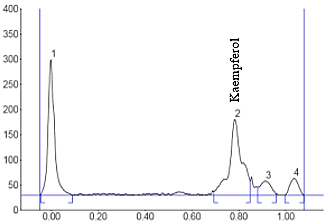 |
| (g) | (h) |

**Figure S4:** Peak chromatogram band of *G. globosa* methanol and chloroform extract using the solvent system toluene-ethyl acetate-glacial acetic acid (30:40:4) (a-d) Methanol extract and (e-h) Chloroform extract of Control, *R. intraradices*, *F. mosseae*, *R. intraradices* + *F. mosseae* respectively.

| 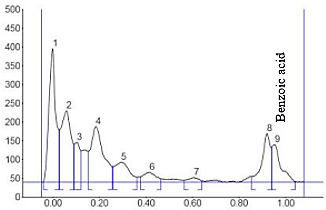 | 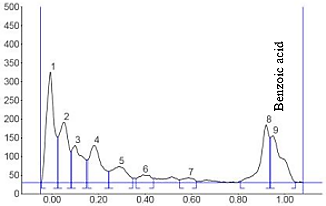 |
| --- | --- |
| (a) | (b) |
| 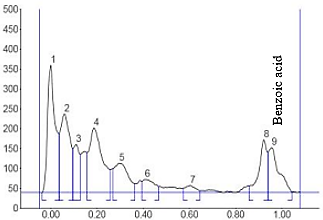 | 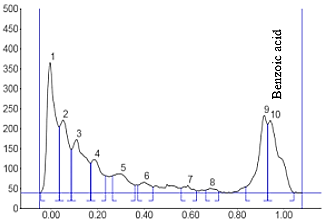 |
| (c) | (d) |
| 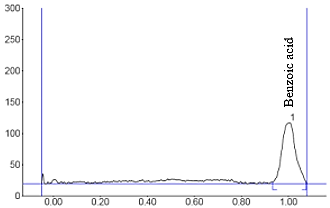 | 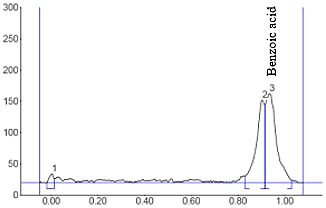 |
| (e) | (f) |
| 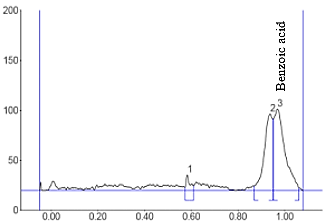 | 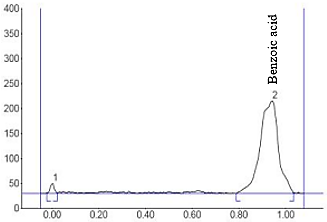 |
| (g) | (h) |

**Figure S5:** Peak chromatogram band of *G. globosa* methanol and chloroform extract using the solvent system toluene-acetone-formic acid (4.5: 4.5: 1) (a-d) Methanol extract and (e-h) Chloroform extract of Control, *R. intraradices*, *F. mosseae*, *R. intraradices* + *F. mosseae* respectively.

| 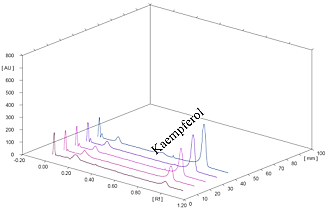 | 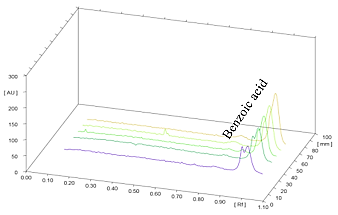 |
| --- | --- |
| (a) | (b) |

**Fig. S6.** HPTLC chromatogram of (a) kaempferol and (b) benzoic acid at 254 nm.
